# Data on medical technology: A new set of variables

**DOI:** 10.1016/j.dib.2020.105534

**Published:** 2020-04-11

**Authors:** Elisabet Rodriguez Llorian, Janelle Mann

**Affiliations:** Department of Economics, University of Manitoba, Canada

**Keywords:** Medical technology, Drugs, Clinical trials

## Abstract

This article provides and describes a database containing three different variables for medical technology. To capture the various dimensions of medical technology, the set of variables covers not only drugs and devices but also general advances in medical knowledge. Information was extracted and processed from the Drug Canada Product Database, and the National Institute of Health. Variables are extracted from 1997 to 2017 and they represent global proxies. The provided data is relevant for healthcare research in various fields of study.

Specifications table

**Table:** 

Subject	Public Health and Health Policy
Specific subject area	Health Economics, Medical Technology
Type of data	Stata data file, Stata code, and Spreadsheet file
How data were acquired	Extraction from public databases
Data format	Raw, Analysed
Parameters for data collection	Start date and end date based on availability of monthly data for all variables.
Description of data collection	Data was extracted via queries executed directly in the databases. Some of the variables were constructed using variables from several database tables.
Data source location	(1) Drug Canada Product Database, Health Canada, Canada (2) National Library of Medicine, National Institutes of Health, United States (www.clinicaltrials.gov)
Data accessibility	With the article

## Value of the data

• The new set of variables for medical technology introduced here are conceptually attractive and add depth to existent measures. While clinical trials successfully represent general developments in medical knowledge, the drug variables capture new ready to use technology.
• This data is particularly relevant to academics in fields of health economics, population health and public policy, as well as to professionals in healthcare policy, planning, investment, and management.
• Variables can be incorporated into empirical research involving medical technology. This could include, for example, work related to innovation, drivers of healthcare expenditure, technology assessment and adoption rates, among others.
• Provided descriptive statistics facilitate data interpretation for explorative studies and illustrative insights.
• Accurate variables for medical technology are crucial when making informed decisions on cost-effective technology that could increase overall quality and specialized support in healthcare services.

## 1 Data description

This paper compiles three proxies for medical technology. As defined by the World Health Organization, medical technology comprises every “application of organized knowledge and skills in the form of medicines, medical devices, vaccines, procedures and systems developed to solve a health problem and improve quality of life” [1]. The new proxies (variables) for medical technology presented here were initially compiled to complement and also overcome some of the limitations of the existent proxies such as expenses on research and development. The validity of the dataset now made available goes beyond a health context and can be extended to research involving medical technology from a variety of fields and approaches.

All variables presented here use data from publicly accessible sources. The code made available with this article can be used to extend the series when updated information becomes available. The time series of over 250 data points (monthly information from 1997 to 2017) allows the researcher to further investigate their relationships and dynamics using a variety of time series techniques and prediction models.

The first variable is a count of completed clinical trial recruitments (CTR) per month. This was obtained from the National Library of Medicine at the National Institutes of Health, which contains a database on clinical trials conducted in 204 countries, including the United States.^1^ A clinical trial is defined as, “a research study in which human volunteers are assigned to interventions (for example, a medical product, behavior, or procedure) based on a protocol (or plan) and are then evaluated for effects on biomedical or health outcomes” [2]. This global medical technology proxy is attractive because it includes advances in medical knowledge such as procedures and tests that are at an advanced stage of research in addition to drugs and devices.

The second and third variables are newly-marketed drugs (NMD), and newly-marketed drugs with new active ingredients (NAI). Both were obtained from the Health Canada Drug Product Database, which provides information on drugs approved for use in Canada. NAI is a subset of NMD, which identifies the newly-marketed drugs which are highly innovative. A drug is considered highly innovative if it “contains a medical ingredient not previously approved in a drug by the Minister and that is not a variation of a previously approved medicinal ingredient” [3]. NMD excludes NAI, which allows the simultaneous inclusion of both variables in empirical estimations. Both medical technology proxies overcome some of the disadvantages of using research and development expenditure or patent data since it includes medical technology that is ready to use in the market. Despite NMD and NAI being constructed based on newly-marketed drugs in Canada, this proxy is relevant beyond Canada because pharmaceutical companies are working to have their drugs approved for use globally simultaneously, to fully benefit from patent laws.

A set of files is made available with this article to allow replication. This include Stata files of the raw data, Stata code as well as a Microsoft Excel spreadsheet with the final database. All files were obtained and/or processed in the following order:

1. Stata data files (raw data): set of four files containing selected variables of interest downloaded from the original sources (after specific search queries were executed as explained in the section below).
2. Stata code: code to be executed on the previous raw data to clean the files and combine existent variables to obtain the desired medical technology proxies
3. Excel Spreadsheet: final medical technology proxies as obtained after running the Stata code on the raw data. The spreadsheet also contains modifications to the technology proxies after incorporating depreciation rates (explained below), as well as all tables and graphs obtained to describe the data.

## 2 Experimental design, materials, and methods

Data for CTR, NMD and NAI was collected directly from the websites of the National Library of Medicine and the Health Canada Drug Product Database Drug and the search queries included the following set of parameters:

• For the case of clinical trials, information was obtained from www.clinicaltrials.gov and gathered through the advanced search feature by selecting those studies where *Study Type* was “All studies” and *Recruitment Status* was “Completed”. Note that no other criteria were pre-selected in the advanced feature. Likewise, under the *Show/Hide Column* option, variables explained in Table 1 were extracted. This query resulted in the database “Clinical Trials.dta” attached to this article.

**Table 1:** Databases and variables used.

Stata Database (raw data)	Variables	Description
Clinical Trials	NCT Number	Unique identifier for each clinical trial
	Primary Completion Date	Date that the last participant in a clinical study was examined or received an intervention to collect final data for the primary outcome measure
	Completion Date	Last participant's last visit
	Title	Name of the clinical trial
Status	id	Unique identifier for each drug
	status	Whether the drug was marketed, approved, dormant or canceled
	date	Date when each status was achieved
Drug	id	Unique identifier for each drug
	type	Whether the drug is human, veterinary or disinfectant
Ingredients	id	Unique identifier for each drug
	activeing	Code for each active ingredient
• For NMD and NAI, three tables were downloaded directly from https://www.canada.ca/en/health-canada/services/drugs-health-products/drug-products/drug-product-database/what-data-extract-drug-product-database.html. These contained information on drugs approved for use in Canada and can be found in the website under the names: QRYM_STATUS, QRYM_DRUG_PRODUCT, and QRYM_ACTIVE_INGREDIENTS. Attached to this article these are included as “Status.dta”, “Drug.dta”, and “Ingredients.dta”, respectively. The three databases made available are reduced to the variables of interest included in Table 1.

Table 1 outlines all variables included in the Stata data files attached to this paper after performing the previous queries. These are the ones needed to obtain the technology proxies. Readers can refer to the original sources for consulting and exploring availability of additional information.

From the information extracted, code is provided to process the raw data and obtain our three variables for medical technology. Steps taken to clean the data are outlined below:

• The number of clinical trials was obtained by counting number of trials using the “NCT Number” and their primary completion date. This was used over the variable “completion date” given the high number of missing values in the latter one.
• To construct the variables NMD and NAI all three files (databases) were merged using each drug unique identifier “id”. First, from the “Status.dta” file we extracted drugs under the “marketed” status. (Once a drug is approved, it can become available for sale. After this step, it becomes a “marketed” product. All marketed products are also approved products). The first “date” the drug in question appears under the "marketed" category is chosen because the aim is to select the month a drug was initially marketed. Additional information is included in the database to help track the history of the product. For example, if a subsequent Notice of Compliance is issued for a drug (e.g. if the company filed a submission for a new indication, a change in manufacturer, etc.) it will appear as another “Marketed” and/or “Approved” status in the database at a later date. Second, from the “Drug.dta” file using the “type” variable only human drugs were kept. The number of NMD is then the count of all human drugs by the date they were first marketed. Lastly, from the “Ingredients.dta” file those drugs that are “highly innovative” were identified to obtain NAI. This was done by flagging the first time an active ingredient had been used in a drug (using each's drugs marketed date and “activeing” code). Notice that one drug can have several active ingredients; a “highly innovative” drug requires one or more of its active ingredients to be in use for the first time.

The original data for each of the three medical technology variables are in the form of a flow. They are converted to stock variables using the perpetual inventory method, as shown in the attached excel spreadsheet. The stock constructed series incorporate the obsolescence of medical technology under different depreciation rates. Table 2 shows descriptive statistics of the initial (flow form) and processed (stock form) data. Processed data include a 10% depreciation rate. Series with 5% and 15% depreciation rates are also included in the spreadsheet attached to this article to facilitate sensitivity analysis in applied research. The conversion to a stock variable results in a continuous time series even though some months have no new drug or clinical trial registrations.

**Table 2:** Descriptive statistics.

	Flow variables			Stock variables (10% depreciation)		
	*NAI*	*NMD*	*CT*	*NAI*	*NMD*	*CT*
Mean	3.11	34.16	415.59	27.77	329.96	3919.90
Standard Error	0.12	1.45	22.20	0.31	11	213.60
Median	3	26.50	404	27.34	266.42	3019.66
Standard Deviation	1.84	23.04	352.43	4.84	174.59	3390.83
Sample Variance	3.40	531.01	124,209.77	23.45	30,481.67	11,497,729.31
Kurtosis	0.70	1.87	−1.04	0.44	−1.17	−1.71
Skewness	0.93	1.33	0.34	0.48	0.56	0.15
Range	9	131	1431	31.54	615.91	9003
Minimum	1	4	0	11.65	80.80	5.52
Maximum	10	135	1431	43.19	696.71	9008.52
Sum	719	8609	104,728	6996.79	83,150.82	987,813.95
Count	231	252	252	252	252	252

**Fig. 1:**
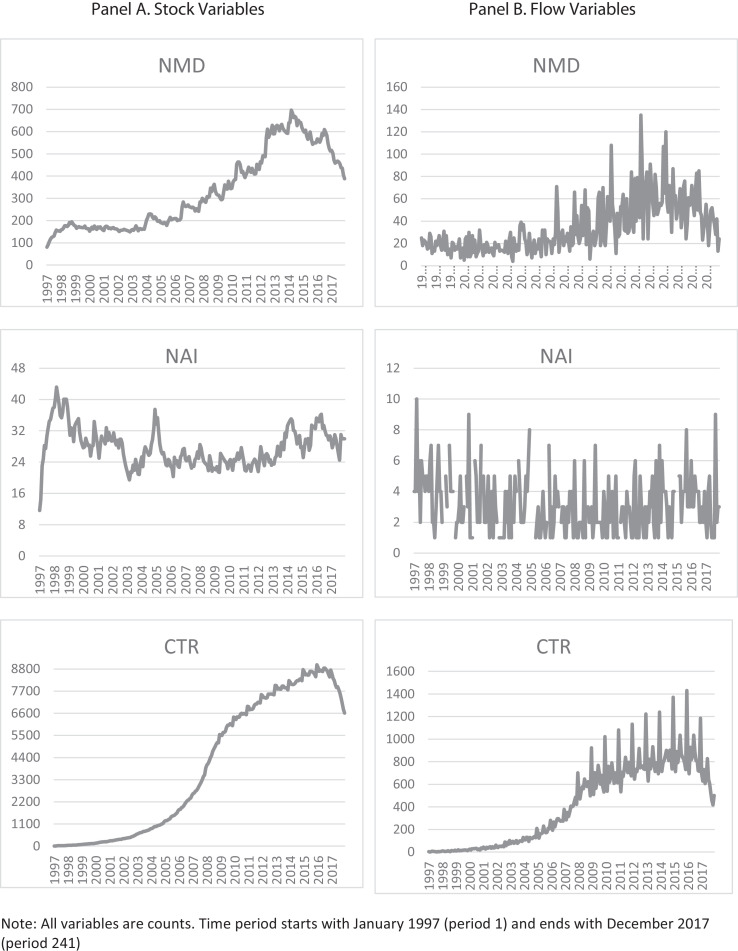
Medical technology variables.

Figure 1 shows a graphical representation of the monthly data (241 data points) for both flow and stock variables.

## Declaration of Competing Interest

The authors declare that they have no known competing financial interests or personal relationships which have, or could be perceived to have, influenced the work reported in this article.

## Appendix Supplementary materials

Image, application 1

## Footnotes

1 We gratefully acknowledge the discovery of this proxy through interdisciplinary dialogue with Dr. Tyler Grant who is the Director of Engineering at Lyndra Therapeutics Inc., and previously a postdoctoral fellow at Langer Lab at MIT.
